# Bright and Multicolor Chemiluminescent Carbon Nanodots for Advanced Information Encryption

**DOI:** 10.1002/advs.201802331

**Published:** 2019-04-15

**Authors:** Cheng‐Long Shen, Qing Lou, Chao‐Fan Lv, Jin‐Hao Zang, Song‐Nan Qu, Lin Dong, Chong‐Xin Shan

**Affiliations:** ^1^ Henan Key Laboratory of Diamond Optoelectronic Materials and Devices School of Physics and Engineering Zhengzhou University Zhengzhou 450052 China; ^2^ Joint Key Laboratory of the Ministry of Education Institute of Applied Physics and Materials Engineering University of Macau Macau 999078 China

**Keywords:** carbon nanodots, chemiluminescence, encryption, energy level alignment, multicolor emission

## Abstract

The various luminescent properties of carbon nanodots (CDs) reveal fascinating applications in several areas. Here, bright and multicolor chemiluminescence (CL) is realized from CDs, whose CL quantum yield can be optimized by adjusting the energy level alignment between the CDs and 1,2‐dioxetanedione intermediate generated from the reaction of peroxalate and hydrogen peroxide. A CL quantum yield of 9.32 × 10^−3^ Einsteins mol^−1^, maximal luminance of 3.28 cd m^−2^, and lifetime of 186.4 s are achieved in red CDs, all of which are the best values ever reported for CDs. As a proof‐of‐concept prototype, a high‐quality information encryption strategy is established via CD based CL imaging techniques by virtue of the high brightness and multicolor CL.

## 1 Introduction

With the advantages of fascinating physicochemical properties and plentiful external morphology, carbon nanomaterials, such as fullerenes, carbon nanotubes, and graphene, have attracted much attention.^1^, ^2^, ^3^, ^4^ Carbon nanodots (CDs) are emerging star of carbon nanomaterials with unique properties, such as good biocompatibility, high photostability, and tunable fluorescence. Because of the above characters, CDs have been considered promising candidate in bioimaging, photocatalysis, optoelectronics, and sensing.^5^, ^6^, ^7^, ^8^, ^9^, ^10^, ^11^, ^12^, ^13^, ^14^, ^15^ However, most of research on CDs are focused on their fluorescence (FL) or electroluminescence (EL) due to the high quantum yield and low toxicity, which are suitable for the application in bioimaging and light‐emitting diodes.^16^, ^17^, ^18^, ^19^, ^20^, ^21^, ^22^ The other luminescent properties of CDs, such as chemiluminescence (CL) are relatively less reported.

CL with light emission induced by energy transfer from chemical reactions, has evoked considerable interest in recent years for its potential applications in chemical detection, bioanalysis, and cold light sources.^23^, ^24^, ^25^, ^26^, ^27^ Although some CL materials, for instance, organic small‐molecule dyes, semiconducting polymer nanoparticles, and inorganic metal nanoparticles, have been employed as CL emitters, most of them encounter serious obstacles in practical application due to their potential biotoxicity, low photostability, high cost, or poor compatibility.^28^, ^29^, ^30^, ^31^, ^32^ Consequently, it is essential to develop environment‐friendly, efficient, and stable CL materials. Notably, a new kind of CL platform has been reported by employing water‐soluble CDs as luminophors or energy acceptor into various chemical reaction systems like HNO_2_‐hydrogrn peroxide (H_2_O_2_), NaIO_4_‐H_2_O_2_, luminol‐KMnO_4_, and Co(II)‐H_2_O_2_.^33^, ^34^, ^35^, ^36^, ^37^, ^38^, ^39^ Nevertheless, very limited progress has been made in CD based CL concerning brightness, efficiency, and multicolor emission because of the following challenges: i) CDs as emitting species in the CL reactions always have large energy interval with the lowest unoccupied molecular orbital (LUMO) of high‐energy intermediate; ii) Excitation wavelength‐dependent photoluminescence (PL) often exists in CDs for CL, dominated by surface or defect states, which not only causes inefficient charge transfer between the CDs and the energy‐rich intermediate but also limits the radiative recombination rate of the excited CDs in the CL systems. Hence, developing high‐efficiency and multicolor CD‐based CL system remains a major challenge.

In this study, CDs with blue, green, and red fluorescence have been synthesized in solvothermal method employing citric acid and urea as precursors by tuning the degree of graphitization and size of conjugated sp^2^‐domains under different solvent conditions.^11^, ^20^ With the merit of the bandgap emissions and energy level alignment between the CDs and the energy‐rich intermediate, multicolor CL systems have been constructed from the CDs through the energy transfer from the chemical reaction of peroxalate and H_2_O_2_. The red CD based CL systems exhibit a maximal quantum yield (QY) of 9.32 × 10^−3^ Einsteins mol^−1^, luminance of 3.28 cd m^−2^, and lifetime of 186.4 s, all of which, to the best of our knowledge, address the best values of previous CDs based CL systems. Based on the bright and multicolor CL of the CDs, we develop a universal application, where high quality of information and pattern encryption are demonstrated as a high level of information security and anticounterfeiting.

## 2 Result and Discussion

### 2.1 Synthesis and Characterization of Multicolor Emissive CDs

**Figure 1:**
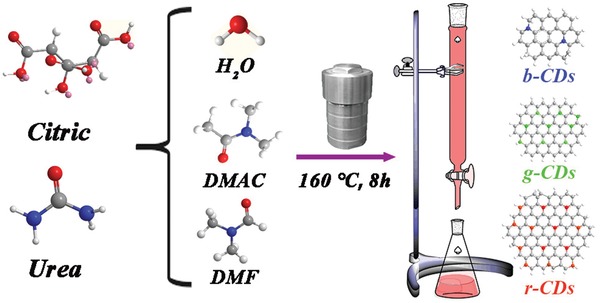
Schematic illustration of the preparation process of the b‐, g‐, and r‐CDs.

**Figure 2:**
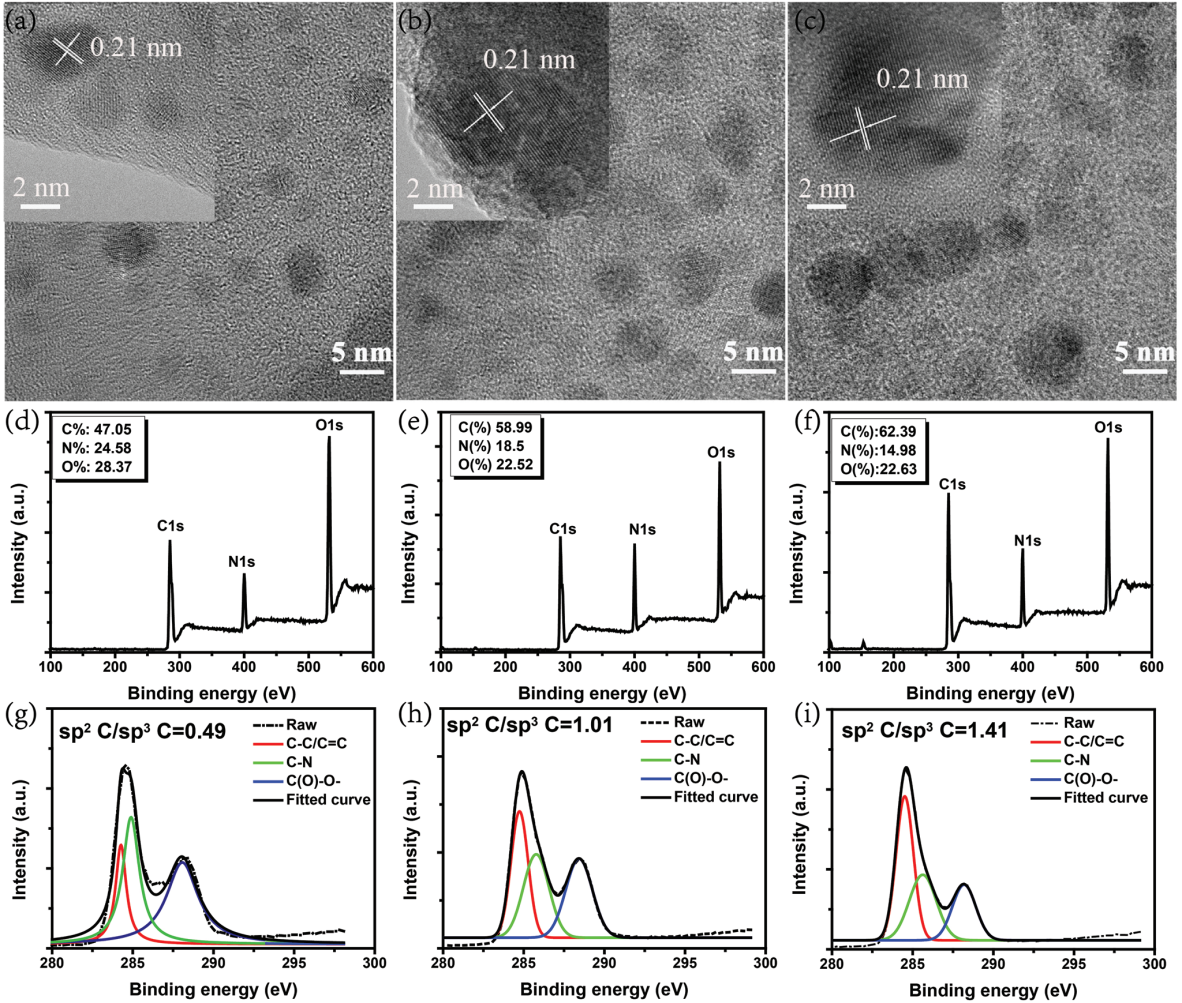
TEM images of the a) b‐CDs, b) g‐CDs, and c) r‐CDs. The insets are the HRTEM images of the corresponding CDs. Survey XPS spectra of the d) b‐CDs, e) g‐CDs, and f) r‐CDs. The insets are the elemental atomic contents estimated from the XPS. High‐resolution XPS spectra of the C1s for g) b‐CDs, h) g‐CDs, and i) r‐CDs.

The multicolor emissive CDs have been synthesized using citric (1 g) and urea (2 g) reacted at 160 °C for 8 h from one‐step solvothermal strategy in 10 mL polar protic water, polar aprotic dimethylacetamide (DMAC), and dimethylformamide (DMF), respectively, as illustrated in **Figure** **1** and in the Experimental Section. The resulting solvents were purified via silica column chromatography using DMF as the eluent, and then the as‐prepared CDs were precipitated with absolute ethyl alcohol and collected by vacuum drying at 60 °C. According to the emission colors, the corresponding produces were named as b‐CDs, g‐CDs, and r‐CDs, respectively. Transmission electron microscopy (TEM) was employed to investigate the morphologies of the three kinds of CDs. As illustrated in **Figure** **2**a,c, the b‐, g‐, and r‐CDs display broad particle size distributions with average diameters of around 3.2, 4.1, and 6.7 nm (Figure S1, Supporting Information). Their high‐resolution TEM (HRTEM) images reveal that all these three kinds of CDs show a well‐resolved lattice spacing of 0.21 nm, which corresponds to the (100) crystallographic plane of graphitic carbon, indicating their high degree of crystallinity and similar core structures.

The chemical composition and functional groups of the three kinds of CDs have been investigated by Fourier transform infrared (FT‐IR) and X‐ray photoelectron spectroscopy (XPS). Figure S2 (Supporting Information) shows similar FT‐IR spectra of the three kinds of CDs, indicating the existence of similar chemical compositions. The peaks at 3417 and 3199 cm^−1^ can be attributed to the stretching vibration of O—H and N—H, and the peaks at 2930 and 1395 cm^−1^ to the stretching and bending vibration of sp^3^ C—H (C—C—H), respectively. And the peaks at 1674 and 1593 cm^−1^ to the stretching and skeletal vibration of sp^2^ C—H (C=C—H), respectively. Comparing the three kinds of CDs, the amount of sp^3^ C—H decreases gradually and the amount of sp^2^ C—H increases gradually from b‐CDs, g‐CDs, to r‐CDs, which means that the degree of graphitization increases from b‐CDs, g‐CDs, to r‐CDs. The survey XPS spectra (Figure 2d–f) of the three kinds of CDs reveal the same composition of C (284.0 eV), N (400.0 eV), O (532.4 eV), and Si (100 eV) elements, which is consistent with the FT‐IR analysis. The atomic ratios of oxygen‐to‐carbon (O/C) slightly decrease from 0.60, 0.38 to 0.36 for b‐, g‐, and r‐CDs, which are distinctively lower than that in the precursor citric acid (0.92) and urea (0.93), which might be due to the increase of dehydrated and carbonized degree of the precursors in the solvothermal process. In this work, chromatographic column was used to purify the CDs and the silica gel is used as the filler of chromatographic column. Therefore, there may be some impurities like silica gel particles in the CDs. As shown in Figure 2d–f, the peak at around 100 eV can be attributed to Si 2p spectra (100.1 eV) and the peak at around 160 eV to Si loss spectra (167.6 eV), which should be caused by residual silica gel in the CD powder for XPS measurement. The high‐resolution XPS C1s spectra are shown in Figure 2g–i. The C1s envelope of the three kinds of CDs can be deconvoluted into three Gaussian peaks corresponding to sp^2^ C (C—C/C=C) at 284.6 eV, sp^3^ C (C—N) at 285.6 eV, and (C=O)—O at 288.2 eV, respectively. It clearly demonstrates that the content of sp^2^ hybrid carbon atoms increase significantly from 0.33 to 0.59 for b‐, g‐, and r‐CDs, which further illustrates the increase in dehydration and carbonization degree of the CDs with longer emission wavelength. Moreover, the deconvoluted high‐resolution XPS spectra for N1s and O1s indicate that the three kinds of CDs contain same kinds of O‐ and N‐containing groups, which agrees well with the above FT‐IR data (Figure S3, Supporting Information). In addition, the content of C=O obviously decreases with the increase in degree of graphitization from b‐CDs, g‐CDs to r‐CDs owing to the dehydration process. The structural information of the CDs has been analyzed by X‐ray diffraction (XRD) and Raman spectroscopy (Figures S4 and S5, Supporting Information). The XRD patterns of the CDs exhibit a broad peak at 23° corresponding to (002) planes of crystallographic structure.^40^, ^41^, ^42^ And the Raman spectra of the CDs present a large intensity ratio of crystalline G band (1580 cm^−1^) and disordered D band at (1350 cm^−1^) of about 0.9–1.1, which further confirms the high degree of crystallinity of the CDs as determined by HRTEM. Hence, the similar chemical constituents and structure reveal that the optical properties of the CDs are largely associated with different degree of graphitization and size of sp^2^‐domains in the CDs.^11^, ^20^, ^41^, ^42^, ^43^, ^44^, ^45^, ^46^, ^47^

### 2.2 Bright Multicolor Fluorescent and Chemiluminescent CDs

**Figure 3:**
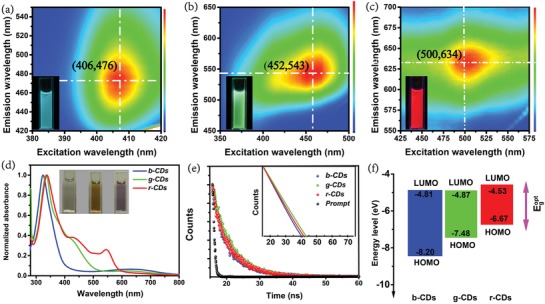
Excitation‐emission matrices of the a) b‐CDs, b) g‐CDs, and c) r‐CDs. The insets are the fluorescence image of the CDs under 365 nm excitation. d) UV–vis absorption spectra of the b‐, g‐, and r‐CDs in aqueous solution. The insets are the fluorescence image of the CDs under sunlight. e) Time‐resolved decay spectra of the b‐, g‐, and r‐CDs in aqueous solution. f) Schematic illustration of the energy level distribution of the b‐, g‐, and r‐CDs.

The photophysical properties of the three kinds of CDs are shown in **Figure** **3** and **Table** **1**. The CDs exhibit bright blue, green, and red luminescence under 365 nm UV light illumination, and the emission peaks are located at about 476, 543, and 634 nm for the b‐, g‐, and r‐CDs, respectively (Figure 3a–c). The excitation‐emission matrices of the CDs indicate that the emission centers are almost immobile and ranged with each maximum peak for the three kinds of CDs in a wide spectral coverage, indicating that the emission of the CDs are all excitation‐independent. The UV–vis absorption spectra of the three kinds of CDs present a strong absorption band at around 330 nm, as indicated in Figure 3d, which derives from the n–π* transition of C=O and conjugated C—N. For the g‐ and r‐CDs, another strong absorption bands appear at about 430 and 550 nm, which are close to their excitation centers at 452 and 500 nm (Figure S6, Supporting Information). Besides, the excitation and emission centers redshift gradually along with the redshift of the excitonic absorption bands, implying that the multicolor emissions originate from the band‐edge transition in the CDs. Furthermore, the time‐resolved decay spectra of the three kinds of CDs can be well fitted with a monoexponential function. And lifetimes of 4.8, 5.5, and 5.2 ns for the b‐, g‐, and r‐CDs have been obtained, which also verifies the near‐band‐edge emissions of the three kinds of CDs, as shown in Figure 3e and Table 1. The PL QYs are determined to be 48%, 33%, and 22% for the b‐, g‐, and r‐CDs, respectively. The decrease in the PL QYs of the CDs with longer emission wavelength might be ascribed to the weakening ability of defect passivation from electron‐donating amino groups along with the increased size of the CDs. In addition, the energy level distribution of the CDs has been also calculated using the optical absorption spectra and ultraviolet photoelectron spectroscopy (UPS) (Figure S7 and Table S1, Supporting Information). The bandgap energies of the CDs can be obtained according to the equation *E*_g_ = 1240/λ_edge_, where λ_edge_ can be fitted by the first excitonic absorption bands of the CDs. For the b‐, g‐, and r‐CDs, the bandgap energies reduce from 3.13 to 2.08 eV, which might result from the increase in the degree of graphitization and the size of the sp^2^‐domains. The result of the UPS spectra reveals the highest occupied molecular orbital (HOMO) levels are −8.20, −7.48, and −6.67 eV for the b‐, g‐, and r‐CDs, respectively.^48^, ^49^ Combined with the results of the bandgap energies, the LUMO levels of the b‐, g‐, and r‐CDs have been calculated at about −5.07, −5.02, and −4.59 eV, respectively. A schematic illustration of the energy level distribution of the CDs is shown in Figure 3f. The above results reveal the emission of the CDs can be modulated by changing the degree of graphitization and size of the conjugated sp^2^‐domains.

**Table 1:** Photophysical characteristics of the b‐, g‐, and r‐CDs excited by the 365 nm line of a mercury lamp

Samples	λ_em_ a) [nm]	*Φ*_F_ b)	CIEc)	τd) [ns]
b‐CDs	476	0.48	(0.19, 0.24)	4.8
g‐CDs	543	0.33	(0.28, 0.43)	5.5
r‐CDs	634	0.22	(0.55, 0.36)	5.2

^a)^ PL maximum peak

^b)^ PL quantum yield

^c)^ Commission Internationale de L'Eclairage coordinate

^d)^ PL lifetime.

**Figure 4:**
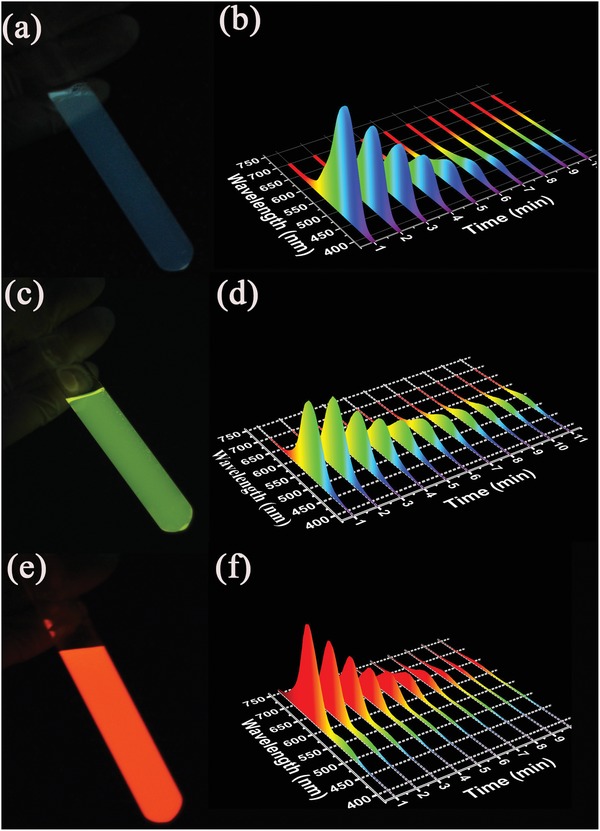
The photograph and decay spectra of the CL from a,b) the b‐CDs, c,d) g‐CDs, and e,f) r‐CDs.

The tricolor bandgap emissive CDs prompt us to exploit their applications in CL luminophors. A conventional peroxalate‐H_2_O_2_ system is employed for the fabrication of CDs‐based multicolor CL. In this system, a hydrophobic oxalate, bis(2,4,5‐trichloro‐6‐carbopentoxyphenyl) oxalate (CPPO) as a chemiluminescent substrate is used for reacting with H_2_O_2_ to generate photons. When the b‐, g‐, and r‐CD solutions are added into the mixture of CPPO and H_2_O_2_, bright, persistent, and multicolor CL can be seen by naked eyes. The b‐, g‐, and r‐CDs demonstrate maximum CL emissions at about 468, 526, and 631 nm, as shown in (**Figure** **4**a–f), which are in good agreement with their PL emission peaks (**Table** **2**). The Commission Internationale de L'Eclairage (CIE) coordinates of the CL emission of the b‐, g‐, and r‐CDs are (0.19, 0.23), (0.34, 0.51), and (0.58, 0.41), corresponding to blue, green, and red emissions, respectively (Figure S8, Supporting Information). The CL decay spectra of the three kinds of CDs keep consistent with their corresponding steady‐state PL spectra, implying the emission of the CD‐based CL system originated from near‐band‐edge transitions of the CDs under the chemical excitation. This may drastically promote the radiative recombination rate of the excited CDs in the CL systems, and then enhance the CL performance. Interestingly, the maximal CL luminance (*L*_max_) of the b‐, g‐, and r‐CDs are 1.67, 2.88, and 3.28 cd m^−2^, respectively, which are the best performance ever reported for CDs‐based CL systems, as shown in Table 2 and Table S2 (Supporting Information). And the CL QYs have been calculated to be 6.60 × 10^−4^, 2.52 × 10^−3^, and 9.32 × 10^−3^ Einsteins mol^−1^ for the b‐, g‐, and r‐CDs using lucigenin as the reference, respectively (Figures S9 and S10, Supporting Information). The CL QYs of the r‐CDs are amongst the highest values ever reported even in all the inorganic nanocrystal based CL systems (Table S2, Supporting Information). Furthermore, the lifetime of the CL were about 178.8, 245.9, and 186.4 s for the b‐, g‐, and r‐CDs when enough CPPO and H_2_O_2_ are supplied in the cold illumination, and the difference might be owing to the large disparity in chemical polarity between the hydrophilic CDs and the hydrophobic peroxalate (Table 2 and Figure S11, and Movies S1–S3, Supporting Information). Moreover, the three kinds of CDs have a good biocompatibility and very low cytotoxicity even at a concentration of 100 µg mL^−1^, and nearly 80% viability for Hela cells has been obtained by incubating the three kinds of CDs for 24 h (Figure S12, Supporting Information), which are beneficial for their future applications.

**Table 2:** Chemiluminescence characteristics of the b‐, g‐, and r‐CDs into the mixed solution of CPPO and H_2_O_2_ in ethyl acetate

Samples	λ_em_ a) [nm]	*L*_max_ b) [cd m^−2^]	*Φ*_C_ c) [Einsteins mol^−1^]	CIEd)	τe) [s]
b‐CDs	468	1.67	6.60 × 10^−4^	(0.19, 0.23)	178.8
g‐CDs	526	2.88	2.52 × 10^−3^	(0.34, 0.51)	245.9
r‐CDs	631	3.28	9.32 × 10^−3^	(0.58, 0.41)	186.4

^a)^ CL maximum peak

^b)^ Maximal CL luminance

^c)^ CL quantum yield

^d)^ Commission Internationale de L'Eclairage coordinate

^e)^ CL lifetime.

**Figure 5:**
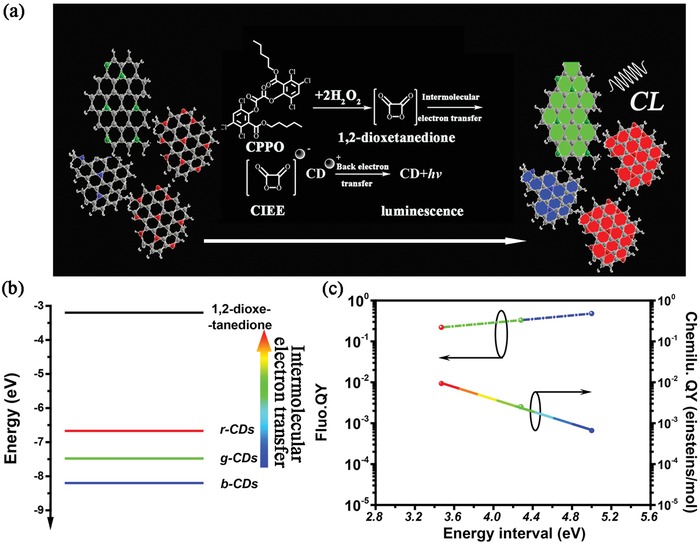
a) Schematic illustration of the chemically initiated luminescence mechanism of the multicolor CDs. b) The LUMO level of 1,2‐dioxentanedione and the HOMO levels of the three CDs. c) The PL and CL QYs of the three CDs as a function of the energy intervals between the LUMO of 1,2‐dioxentanedione and the HOMO of CDs.

The mechanism of the CD‐based efficient and multicolor CL might be due to the chemically initiated electron exchange luminescence (CIEEL), as shown in **Figure** **5**a.^28^, ^50^ The CIEEL is suggested to involve a sequence of steps. First, the oxidation reaction of peroxalate compound (CPPO) and H_2_O_2_ spontaneously yield an energy‐rich intermediate–1,2‐dioxetanedione. Second, the intermolecular electron transfer from the CDs to the intermediate will evoke the cation CD radicals and the anion carbon dioxide radicals. Third, the electron transfers back from the anion to the cation, engendering excited CDs. Finally, the excited CDs relax from excited state to ground state by releasing photons, leading to the bright and multicolor CL. And the electron exchange luminescence has been supported by the ESR and time‐resolved decay spectra (Figures S13–S15, Supporting Information). Besides the bandgap transitions in the CDs, the energy level alignment between the luminescence reporter (CDs) and the CL intermediate (1,2‐dioxetanedione) is another key factor contributing to the high CL performance. The energy alignment can promote the intermolecular electron transfer from the CDs to 1,2‐dioxetanedione. Degree of freedom for such an intermolecular electron transfer process can be qualitatively evaluated according to the energy interval between the HOMO of the CDs and the LUMO of energy‐rich 1,2‐dioxetanedione. Among the three kinds of CDs, the HOMO of the r‐CDs is the closest to the LUMO of the 1,2‐dioxetanedione, which implies that largest degree of freedom for the electron transfer process can be obtained in the r‐CDs, bringing about the highest CL QY (Figure 5b). As shown in Figure 5c, the CL QYs of CDs are inversely proportional to the energy interval between the HOMO of the CDs and LUMO of the intermediates, while their PL QYs do not follow in such a way. Because the CL QYs of CDs are mainly dominated by the degree of freedom for the electron transfer process, namely, the energy level alignment.

### 2.3 Information and Pattern Encryption Based on the CL of the CDs

Growing levels of information interception have aroused a serious hazard to individual and national security. The traditional confidential manners are very difficult to meet the ever‐increasing needs of information security. The advantages of bright, multicolor, and nontoxic CD based CL may open perspectives for information security applications.

**Figure 6:**
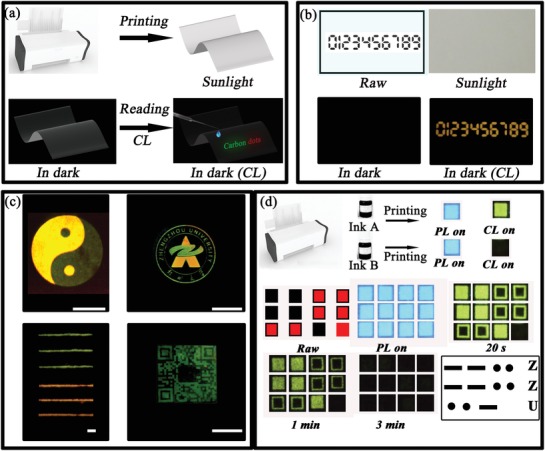
a) Schematic illustration of the CL based information encryption and decryption. b) Photographs of commercially available printing paper with printed encrypted Arabic numerals using r‐CDs as ink under sunlight, the images before and after spraying with CPPO/H_2_O_2_ solution in dark. c) Different paper‐based chemiluminescence images: An image of Eight Diagrams printed using r‐CDs and g‐CDs as ink after spraying with CPPO/H_2_O_2_ solution (Scale bar = 5 cm), a colorful printing of badge of Zhengzhou University using a cartridge filled with g‐CDs, and r‐CDs (Scale bar = 1 cm), an image of green (the upper) and red (the below) lines with the point size of 0.5 printed using cartridges filled with r‐CDs and g‐CDs and the QR code image with g‐CDs using cartridges filled with g‐CDs (Scale bar = 2 cm), respectively. d) Burn‐after‐reading information encryption process based on CD based CL system and the images of the Morse code printed with g‐CDs (Ink A) and g‐CDs/NaAc (Ink B) as inks under UV light and after spraying CPPO/H_2_O_2_.

The promising applications of the CDs based CL systems in information encryption have been illustrated in **Figure** **6**a. In the encryption process, some Arabic numerals are printed onto a Whatman filter paper with r‐CD ethanol solution as ink. The printed information cannot be observed under sunlight illumination conditions (Figure 6b). For decryption, r‐CD based CL is employed to highlight the encrypted Arabic numerals. That is, when the mixture solution of CPPO and H_2_O_2_ is sprayed onto the paper, the bright luminescence appears on the paper and the encrypted information can be observed clearly with naked eyes. Beyond that, various complicated and even multicolor patterns (square, pattern, badge, lines, and letters) can also be encrypted by using commercially available cartridges filled with g‐, and r‐CD ethanol solution (Figure 6c and Figures S16–S22, Supporting Information). For example, a high‐definition and luminous image of Eight Diagrams has been achieved by our CD based CL using r‐CD and g‐CD ethanol solution as ink. Moreover, green‐and‐red images of a badge of Zhengzhou University and lines present distinguishable colors on the paper. Meanwhile, the lines and letters in size 5 could be easily observed with no need for external excitation source like harmful UV radiation (Figure S20, Supporting Information). In addition, replacing traditional inks by the biocompatible CDs as ink, encrypted QR code and fingerprints could also be obtained on the paper, revealing that the CD can also potentially employed as an advanced anticounterfeiting ink using the CL imaging method (Figure 6c and Figure S22, Supporting Information). Furthermore, a concept of burn‐after‐reading information encryption has also be established from the CD based CL system for the first time. Here, we demonstrate a Morse code, widely used in a communications system, can be decoded only once for retrieving the encrypted information. As shown in Figure 6d, solutions of g‐CDs and the mixture of g‐CDs and CH_3_COONa (NaAc, a catalyst to accelerate the chemical reaction in the oxalate system and largely reduce the lifetime of the CL system to 0.96 s) were used as black (A) and red (B) ink, respectively (Figure S23, Supporting Information). The two solutions present similar reflectance, PL, and CL spectra when printed on the commercially available photo paper except that the CL of g‐CDs/NaAc shows a much lower intensity than that of the g‐CDs (Figures S24–S26, Supporting Information). For the Morse code, the information printed with ink A and B represents “dashes” and “dots” (Figure S27, Supporting Information). Under the UV light, only the similar fluorescent spots can be observed (Figure 6d). Nevertheless, after spraying the mixture solution of CPPO and H_2_O_2_, we can distinguish the bright and dark spots for up to 3 min. And the information can be easily decrypted into “ZZU” by the rules of Morse code. The bright spots are gradually out of sight in dark. These observations demonstrate that this CL imaging technique on paper could provide more options in data encryption and anticounterfeiting.

## 3 Conclusion

In summary, an efficient and multicolor emissive CDs based CL system has been demonstrated, in which blue, green, and red emissive CDs have been introduced as luminophors. These multicolor CDs are solvothermally synthesized with the same precursors by tuning the conjugated sp^2^‐domain size under different solvent conditions. The multicolor CL can be obtained from the CDs via chemically initiated electron exchange luminescence in the chemical reaction of peroxalates and H_2_O_2_. With the help of the bandgap transitions in the CDs and the energy level alignment between the luminescence reporter (CDs) and the CL substrate (1,2‐dioxetanedione), the relevant r‐CDs based CL systems exhibit a maximal QY of 9.32 × 10^−3^ Einsteins mol^−1^, *L*_max_ of 3.28 cd m^−2^ and lifetime of 186.4 s, all of which are the highest values ever reported for CD based CL systems. Moreover, information encryption–decryption process and high‐quality multicolor patterns for advanced anticounterfeiting are achieved via spraying peroxalate and H_2_O_2_ solution onto the CD‐printed paper. Overall, this bright and multicolor CD based CL system holds great promise for the achievement of advanced security protection, which will inspire further development and expand the scope of research and applications of CD based CL.

## 4 Experimental Section

*Synthesis and Purification of b‐CDs, g‐CDs, and r‐CDs*: 1 g citric acid and 2 g urea were dispersed in 10 mL deionized water, *N*,*N*‐dimethylacetamide (C_4_H_9_NO, DMAC), and *N*,*N*‐diethylformamide (C_3_H_7_NO, DMF), respectively. The mixtures were added into Teflon‐lined stainless autoclave (20 mL). Then the sealed autoclave vessels were placed into an electric oven, which was set at 160 °C and hold for 8 h. The resulting solvents were purified via silica column chromatography using DMF as the eluent, and then the as‐prepared CDs were precipitated with absolute ethyl alcohol and collected by vacuum drying at 60 °C for 1 day. The final products were collected for characterizations and further used.

More details about the characterization of materials and data encryption procedure are demonstrated in the Supporting Information.

## Conflict of Interest

The authors declare no conflict of interest.

## Supporting information

Supplementary

Supplementary

Supplementary

Supplementary
