# Supplementary material for: Bright and Multicolor Chemiluminescent Carbon Nanodots for Advanced Information Encryption
Source: Adv Sci (Weinh). 2019 Apr 15;6(11):1802331. doi: 10.1002/advs.201802331 (PMC6548985; doi:10.1002/advs.201802331)
Supplement: Supplementary file 1 — Supplementary [file ADVS-6-1802331-s002.pdf]

Copyright WILEY-VCH Verlag GmbH & Co. KGaA, 69469 Weinheim, Germany, 2019.

ADVANCED SCIENCE
Open Access

Supporting Information

for *Adv. Sci.,* DOI: 10.1002/advs.201802331

Bright and Multicolor Chemiluminescent Carbon Nanodots for Advanced Information Encryption

Cheng-Long Shen, Qing Lou, Chao-Fan Lv, Jin-Hao Zang, Song-Nan Qu, Lin Dong, and Chong-Xin Shan**

Copyright WILEY-VCH Verlag GmbH & Co. KGaA, 69469 Weinheim, Germany, 2019.

Supporting Information

Bright and Multicolour Chemiluminescent Carbon Nanodots for Advanced Information Encryption

Cheng-Long Shen, Qing Lou, Chao-Fan Lv, Jin-Hao Zang, Song-Nan Qu, Lin Dong, Chong-Xin Shan**

EXPERIMENTAL SECTION

Materials.

All the chemicals and solvents were used without further purification.

Characterization.

The surface morphology of the three CDs were characterized by a field emission transmission electron microscope (HRTEM, JEOL JSM-IT100). The absorption spectra were measured on a Hitachi U-3900 UV-VIS-NIR spectrophotometer. The fluorescence spectra were measured by a spectrofluorometer (Hitachi F-7000). The fluorescence decay curves were measured by a Horiba FL-322 spectrometer using a 370 nm NanoLED monitoring the emission at 475 nm, 540 nm and 630 nm, respectively. The absolute PL QYs were also measured by a Horiba FL-322 spectrometer with a 365 nm NanoLED as excitation source. X-ray photoelectron spectroscopy (XPS) was measured on a Kratos AXIS HIS 165 spectrometer with a monochromatized Al KR X-ray source (1486.7 eV). Ultraviolet photoelectron spectroscopy (UPS) was obtained on a Thermo ESCALAB 250XI spectrometer. Electron spin resonance (ESR) was measured by a Bruker A300 Electron paramagnetic spectrometer. The Fourier transform infrared spectra (FT-IR) of the CDs were recorded on a Bio-Rad Excalibur spectrometer (Bruker vector 22). The X-ray diffractometer (Panalytical X' Pert Pro) using Cu k_{α} as the irradiation source was used

to obtain the XRD patterns. Raman spectra were carried on a Raman spectrometer (Renishaw inVia). The optical and chemiluminiscence (CL) images were obtained using a Nikon D600 digital camera. The maximal CL luminance of the CDs was measured by using a system comprising a photometer (Minolta Luminance Meter LS-110) during the continuous process of CL. The information was printed on an ink cartridge printer (Epson L310).

Supplementary Figures, Notes, and Tables.

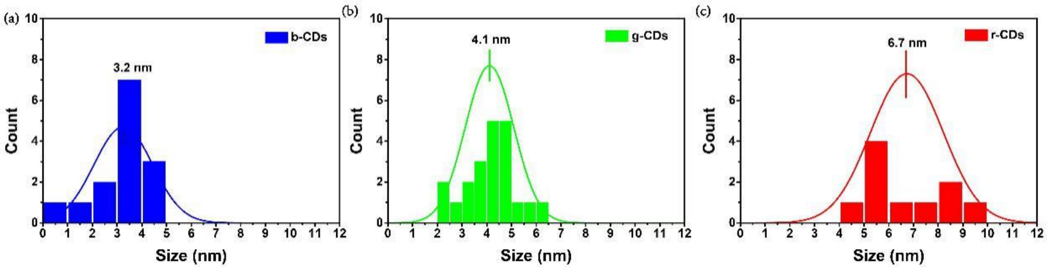

Figure S1. a-c) The size histogram of b- (a), g- (b), and r-CDs (c).

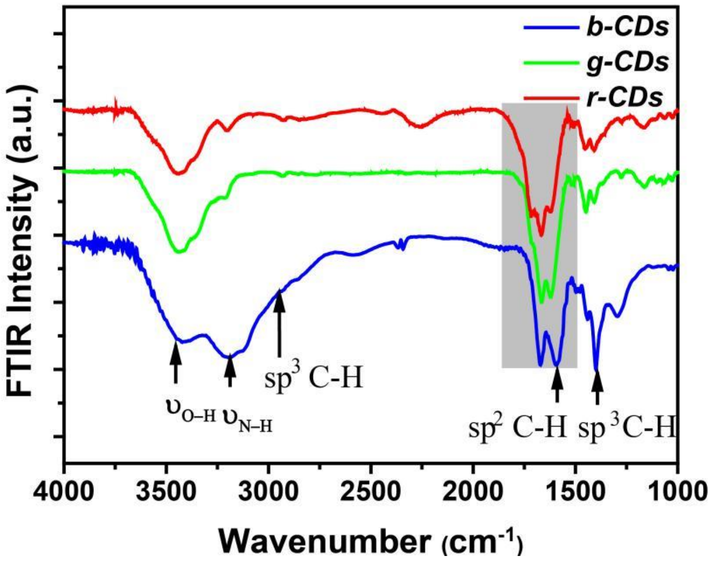

Figure S2. The FTIR spectra of b-, g-, and r-CDs.

Note: The sp2/sp3 ratio has also been measured from the FTIR spectra of the CDs, as indicated in Figure S2. The ratio is 0.89, 1.68, and 2.03 for the b-CDs, g-CDs, and r-CDs, respectively. We note that the ratio derived from the XPS spectra is consistent with that derived from the FTIR spectra, which indicates the different degree of

graphitization of the three kinds of CDs.

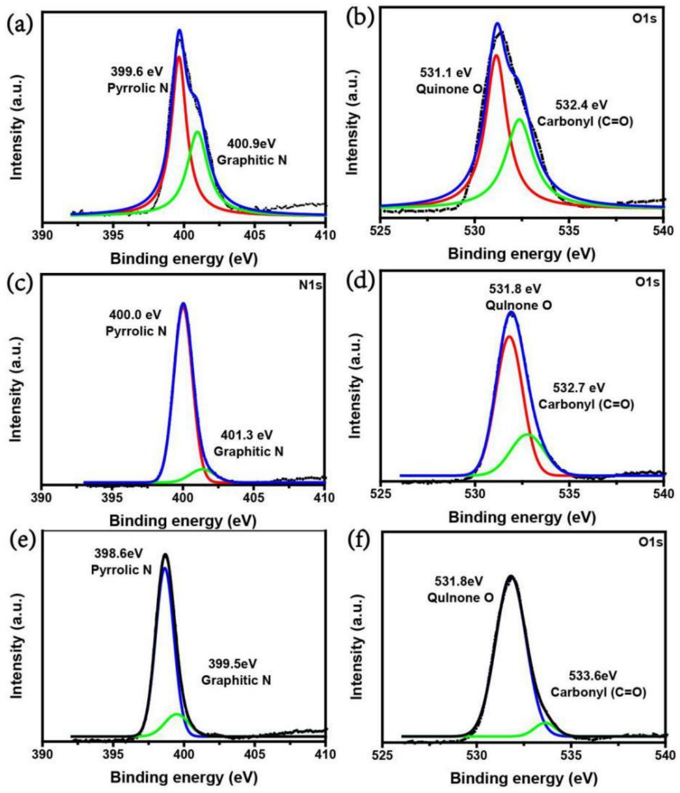

Figure S3. a-f) The N1s (the left) and O1s (the right) spectra of b- (a,b), g-(c,d), and r-CDs (e,f).

Note: The number of the surface oxygen has been measured via XPS, the results of which are shown in Figure S3. The content of C=O obviously decreases with the increase in degree of graphitization from b-CDs, g-CDs to r-CDs owing to the dehydration process.

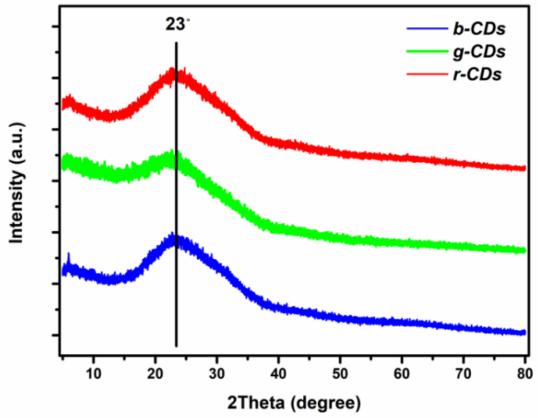

Figure S4. The XRD of b-, g- and r-CDs

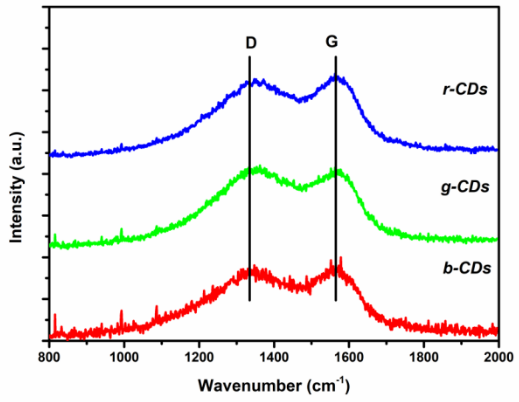

Figure S5. The Raman spectra of b-, g- and r-CDs.

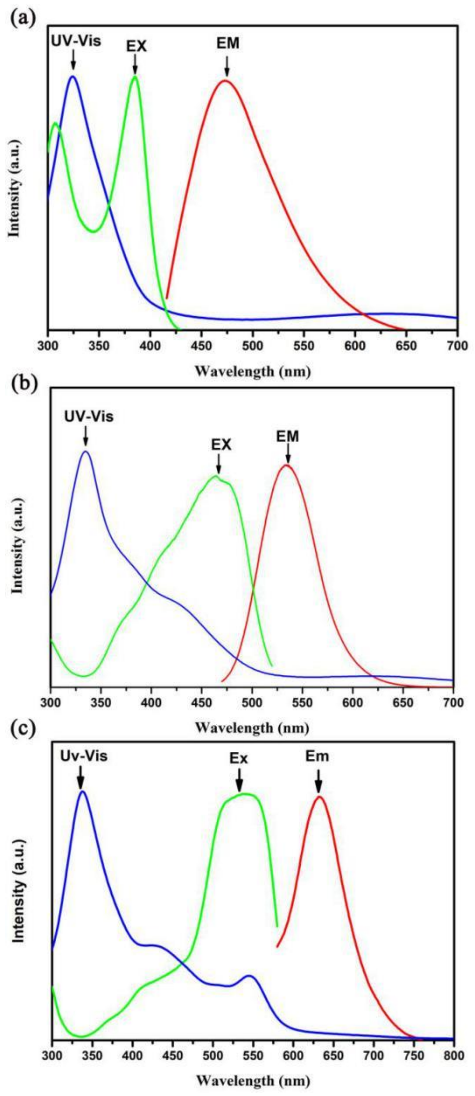

Figure S6. a-c) The UV-Vis (blue), excitation (green) and emission (red) spectra of b- (a), g- (b), and r-CDs (c).

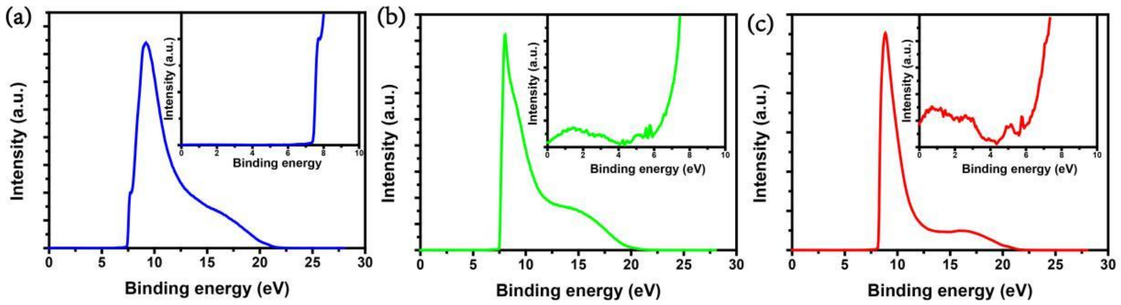

Figure S7. Ultraviolet photoelectron spectroscopy (UPS) data of b- (a), g- (b), and r-CDs (c), respectively

Note: the HOMO and LUMO position of the CDs have been calculated based on the UPS results and the bandgap derived from the absorption bands of the CDs [1].

Table S1. Estimation of the energy levels of b-, g- and r-CDs.

CDs	HOMO(eV)	LUMO(eV)	λ_{edge} (nm)	E_g^{opt} (eV)
b-CDs	-8.20	-5.07	395	3.13
g-CDs	-7.48	-5.02	505	2.46
r-CDs	-6.67	-4.59	595	2.08

Chemiluminescence measurements.

The CL spectra were measured by the Hitachi F-7000 spectrofluorometer with the excitation light source off. 0.5 mL different CDs and lucigenin (3 mg mL^{-1}) ethyl alcohol solution were injected into the mixture of 1 mL bis(2,4,5-trichloro-6-carbopentoxyphenyl) oxalate (0.2 M, CPPO) ethyl acetate solution and 1 mL H_2O_2 (1 M) ethyl alcohol solution. The CL kinetic curves were also measured by the F-7000 spectrofluorometer and collected after 1 min of mixing the solution with 1 min integration.

Chemiluminescence quantum yield (CL QYs) of b-, g- and r-CDs.

The CL QYs of b-, g-, and r-CDs were measured using lucigenin with H_2O_2 as oxidant with a known QY of 8.3×10^{-3} einsteins mol^{-1} at PH=11 according to the previous literatures [2-4]. According to the CL spectra and kinetic curves, the CL QYs were calculated according to the following equations:

$$\phi_{CL} = \frac{Q\times f_{luc}\times f_{photo}}{n}(einsteins/mol) \quad (1)$$

$$f_{luc} = \frac{\phi_{luc}\times n_{luc}}{Q_{luc}} \quad (2)$$

$$f_{photo} = \frac{f(\lambda_s)}{f(\lambda_{luc})} \quad (3)$$

Where ϕ_{CL} is the CL QYs of b-, g-, and r-CDs, Q is the total light emission obtained by integration of emission intensity under time curves. f_{luc} is obtained by measuring the emission kinetics of lucigenin reaction performed in standard conditions. f_{photo} is obtained from the sensitivity at the emission wavelength (λ_{max} =475 nm) of the lucigenin standard, $f(\lambda_{luc})$, and the emission of the b-, g- and r-CDs, $f(\lambda_s)$. n is the number of moles of lucigenin (n_{luc}) or the number of moles of CPPO.

Note: Generally, the fluorescence QY of the CDs is mainly determined by the radiative recombination probability of the excited electron-hole pair. While the chemiluminescence (CL) QY of the CDs is not only determined by the radiative recombination ability of the exciton, but also determined by the electron exchange efficiency between the CDs and energy-rich intermediate. In the CPPO-CDs-H_2O_2 system, the CL QY of the three kinds of CDs can be calculated by the following expression:

$$CL\ QY = Y_1*Y_2*Q_{FL}$$

Where Y_1 is the productivity of intermediate, Y_2 is the productivity of excited CDs by CIEEL between the CDs and intermediate, and the Q_{FL} is the fluorescence QY. The

CL QY is dominated by the electron exchange efficiency between the CDs and the intermediate. The electron exchange efficiency between the CDs and 1,2-dioxetanedione is determined by the CIEEL degree of freedom, which can be qualitatively evaluated according to the energy interval between the highest occupied molecular orbital (HOMO) of the CDs and the lowest unoccupied molecular orbital (LUMO) of 1,2-dioxetanedione. Among these CDs, the HOMO of r-CDs is the closest to the LUMO of 1,2-dioxetanedione, thus the electron exchange between them is most efficient, leading to the highest CL QY.

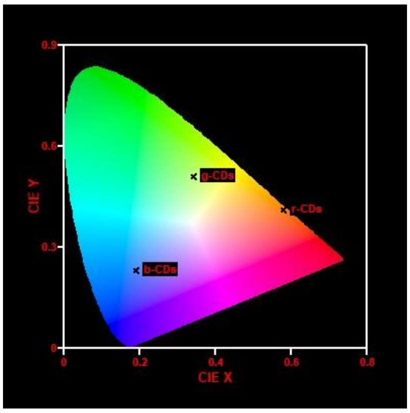

Figure S8. The CIE coordinates of CL illumination based on the b-, g-, and r-CDs.

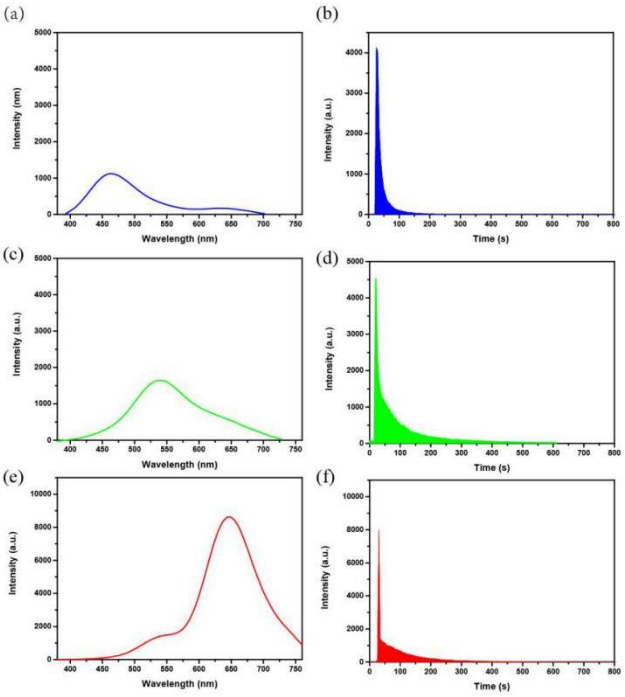

Figure S9. a-f) The CL spectra (the left) and decay curves (the right) of b- (a,b), g-

(c,d), and r-CDs (e,f).

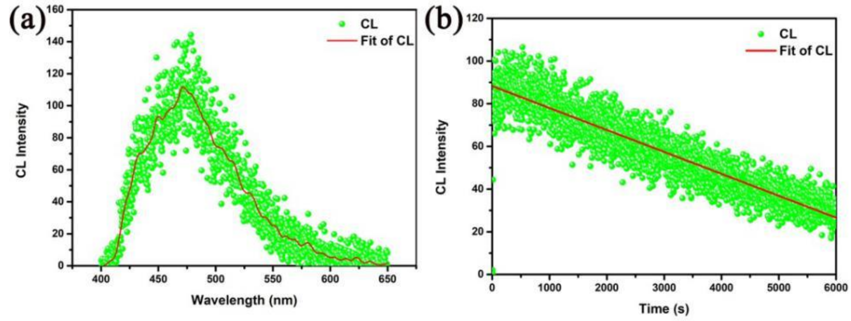

Figure S10. a,b) The CL spectra (a) and decay curves (b) of lucigenin.

Chemiluminescence Illumination.

The CL illumination was established by a similar method used in the commercial light stick. The reaction solution A was prepared by dissolving 4 g CPPO and 5 ml CD (3 mg mL^{-1}) into 95 ml ethyl acetate. The reaction solution B was prepared by dissolving 40 mL H_2O_2 (wt% = 30%) into 60 ml ethyl ethanol. Then, the solution A and solution B were mixed in a test tube, and a continuable phenomenon of CL would appear. The CL could be observed by the naked eyes and could be used for illumination.

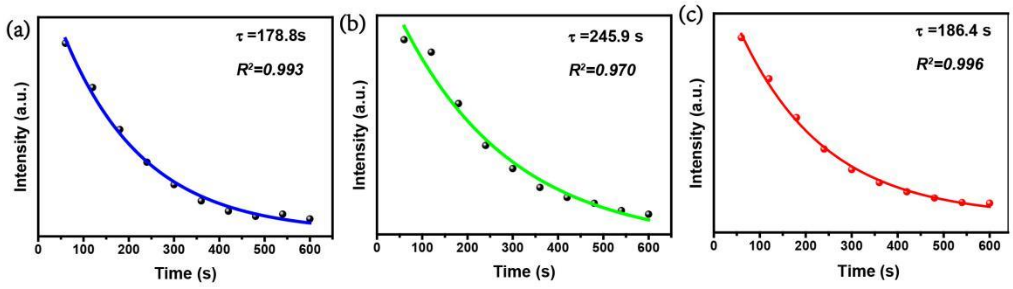

Figure S11. a-d) The fitted curves of CL decay spectra for b-CDs (a), g-CDs , and r-CDs (c).

Cytotoxic Evaluation.

The Hela cells were incubated with CDs aqueous solution at different concentrations (0, 0.8, 4, 20, and 100 μg mL^{-1}) for 24 h at 37 °C. Then, the viability of Hela cell was examined using standard MTT method for assessing the cytotoxicity of the three CDs.

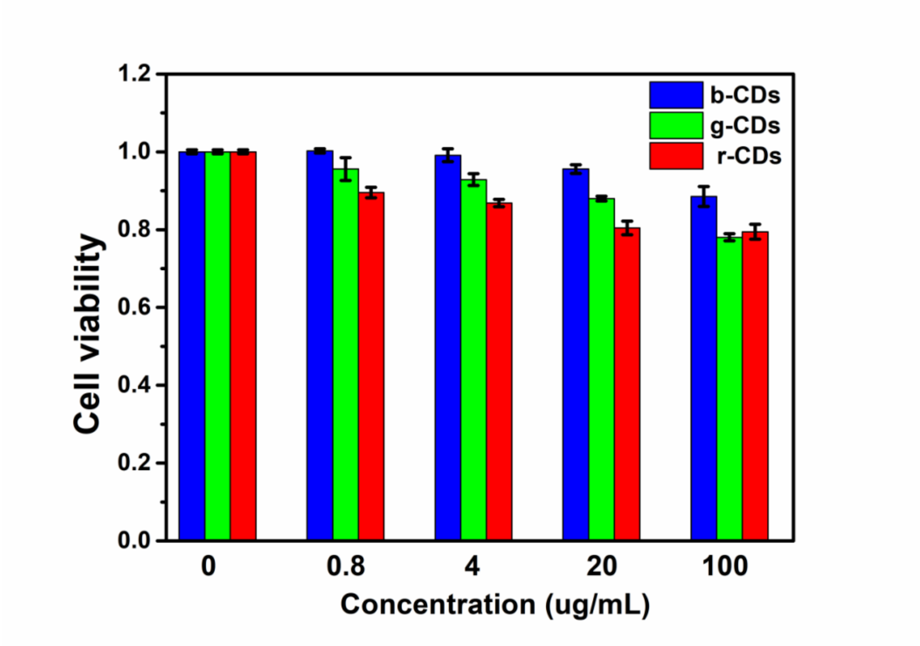

Figure S12. Cell viability of Hela cells after 24 h incubation in the different concentration of the b-, g-, and r-CDs.

Chemiluminescence mechanism.

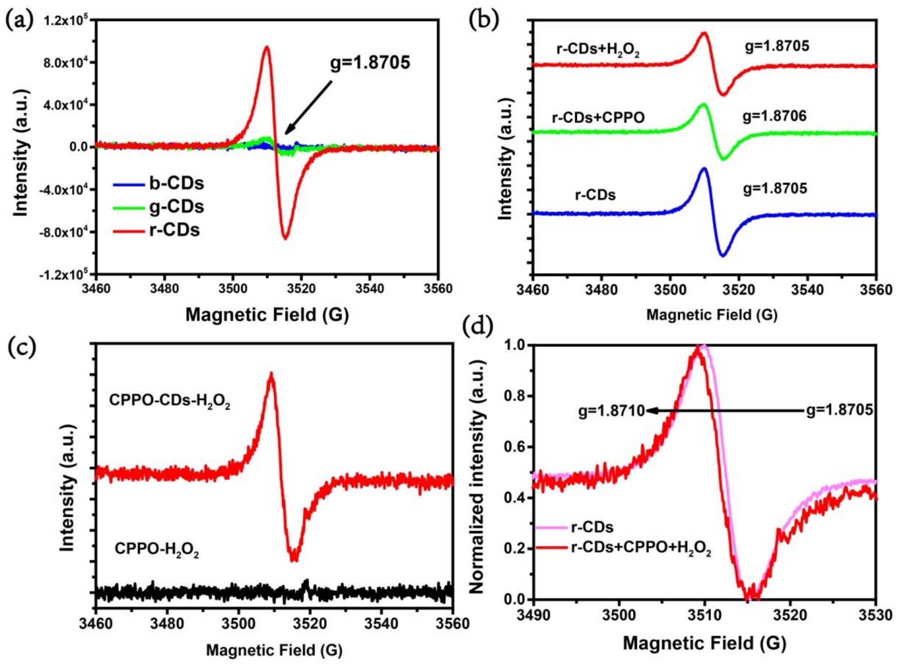

Figure S13. (a) ESR spectra of the b-, g-, and r-CDs; (b) ESR spectra of the CDs, CDs/CPPO, and CDs/H_2O_2; (c) ESR spectra of the CDs in the CPPO-H_2O_2 system with/without the CDs; (d) ESR spectra of the CDs before and after addition of H_2O_2 and CPPO.

Note: ESR method was used to investigate the ground-state properties of luminescent species in the CDs. As shown in Figure S13, the three kinds of CDs show ESR signal at g = 1.8705, which illustrates singly occupied orbit in the ground-state of the CDs. The singly occupied orbit indicates that the CDs can act as electron donors or acceptors during the CL process. The g-value of the CDs after their reaction with CPPO or H_2O_2 shows little shift compared to that of the pristine CDs (see Figure S13b), which verifies that there is no direct interaction between CDs and CPPO or H_2O_2. Moreover, CDs-CPPO-H_2O_2 system also presents a similar singly occupied orbit. Nevertheless, without CDs, the H_2O_2-CPPO system exhibits no ESR signal, implying that the CDs are the main contributor to the CL (see Figure S13c). Furthermore, the g-value of the CDs increases from 1.8705 to 1.8710 after addition of H_2O_2 and CPPO, indicating electron exchange between the singly occupied orbit in the CDs and intermediate during the CL process.

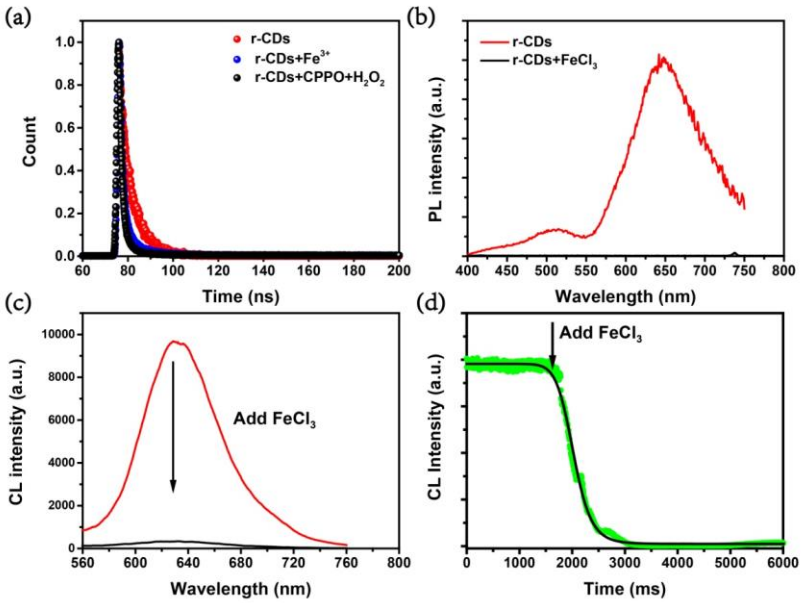

Figure S14. (a) The time-resolved decay spectra of r-CDs, r-CDs after adding Fe^{+3}, and r-CDs after adding CPPO/H_2O_2; (b) PL spectra of r-CDs before and after adding

Fe^{3+}; (c) CL spectra of r-CDs before and after adding Fe^{3+}; (d) CL intensity decay of r-CDs after Fe^{3+}.

Note: The electron transfer between the CDs and intermediate is also investigated by the time-resolved decay spectra. As shown in Figures S14, the time-resolved decay spectra of CDs and CDs after adding CPPO/H_2O_2 can be well-fitted with one-order exponentially decay with a PL lifetime of 5.20 ns and 1.97 ns. The decreased PL lifetime of CDs with the addition of CPPO and H_2O_2 can be attributed to the electron transfer from the CDs to the intermediate derived from the reaction of CPPO and H_2O_2. Furthermore, Fe^{3+}, as an efficient PL quenching agent, has been extensively investigated as an electron acceptor in luminescence systems. As shown in Figures S14a and S14b, the CDs show a decreased PL lifetime and typical PL quenching after the addition of Fe^{3+}, which proves the electron transfer from the CDs to Fe^{3+}. Similarly, the CL of the CDs can also be quenched by Fe^{3+}. As shown in Figures S14c and S14d, the addition of Fe^{3+} in the CDs-CPPO-H_2O_2 system can decrease the CL intensity sharply, which means that the electron transfer from the CDs to the intermediate during the CL process can be destroyed by the Fe^{3+}. Hence, electron transfer from CDs to the intermediate indeed exists in the CL process.

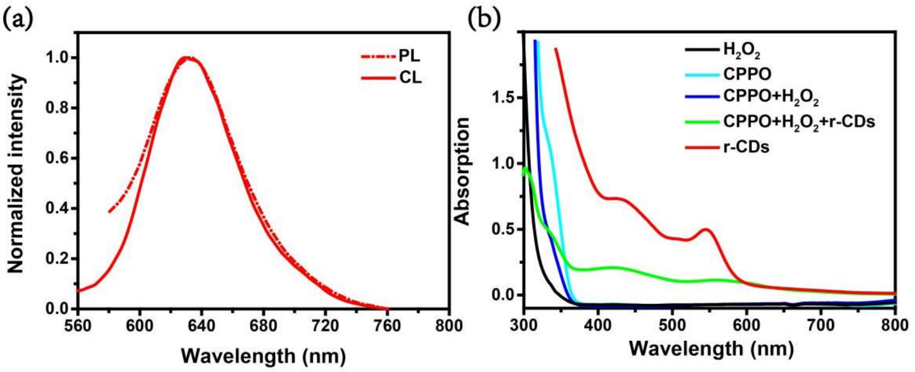

Figure S15. (a) PL and CL spectra of r-CDs; (b) UV-Vis spectra of H_2O_2, CPPO, CPPO/H_2O_2, CPPO/H_2O_2/r-CDs, and r-CDs.

Note: The role of nanoparticles in liquid-phase CL reaction can be as catalysts or

emitters. In order to identify the roles of the CDs in the CPPO-CD-H_2O_2 CL system, CL spectra have been measured by a fluorescence spectrometer with the Xenon lamp turned off. As shown in Figure S15, the CL spectra of the CDs show similar profile with their PL spectra. Moreover, the UV-Vis spectra of the CDs before and after the CL reaction present no new absorption bands, revealing that the CDs are emitters in the CL reaction. Hence, it is reasonable that CL can be attributed to the radiative recombination of the excited CDs though the electron exchange between chemical reaction and CDs.

Data encryption and recording based on the chemiluminescence of CDs.

The method of information encryption and recording was based on the CL of CDs. The common printing paper was used as the substance and the b-, g-, and r-CDs (3 mg mL^{-1} in ethyl alcohol) were used as the blue, green, and red inks. The pattern information was printed by a HP DeskJet 2020 printer with the three CDs as inks. The desired patterns printed onto a piece of CD-coated paper can be read out after spraying a mixture solution of CPPO and H_2O_2 on the paper. Fingerprinting was conducted by pressing a fingertip dipped in a g-CD (3 mg mL^{-1} in ethyl alcohol) on a piece of paper for 5 seconds. An image of fingerprint with bright luminescence could be obtained when the mixture solution of CPPO and H_2O_2 was sprayed on the paper.

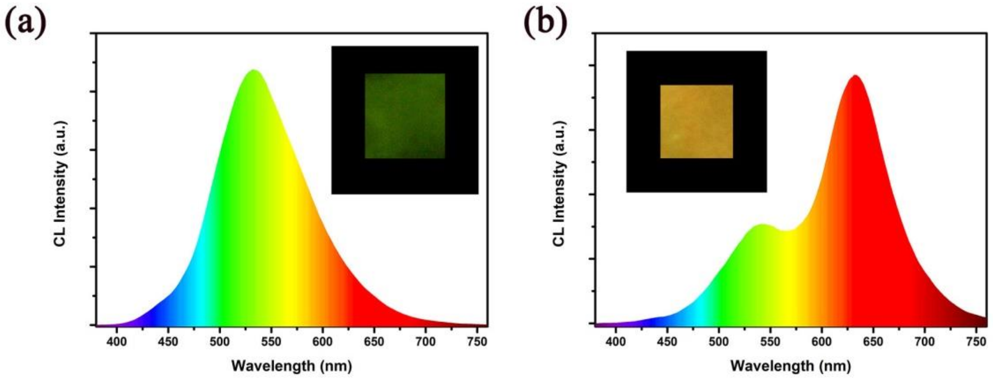

Figure S16. a,b) The spectra of paper-based CL using g- (a) and r-CDs (b) as ink. The inset is the corresponding photographs of the g- (a) and r-CD (b) based CL systems on a filter paper.

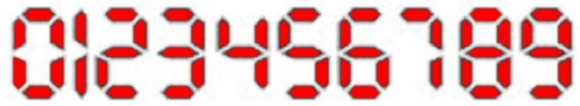

Figure S17. The pattern prototype of Arabic numerals used for printing.

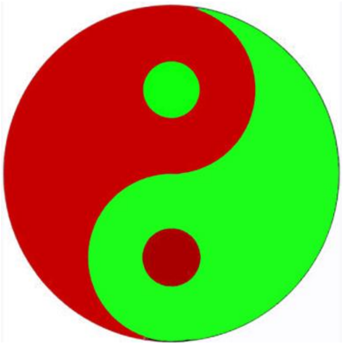

Figure S18. The pattern prototype of Eight Diagrams drawing by us used for printing.

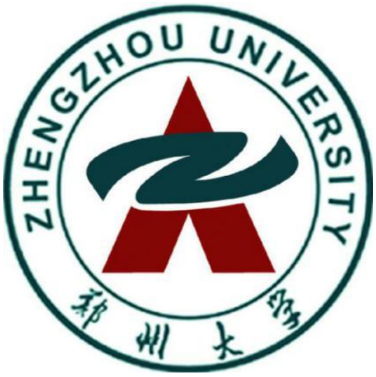

Figure S19. The pattern prototype of badge of Zhengzhou University used for printing.

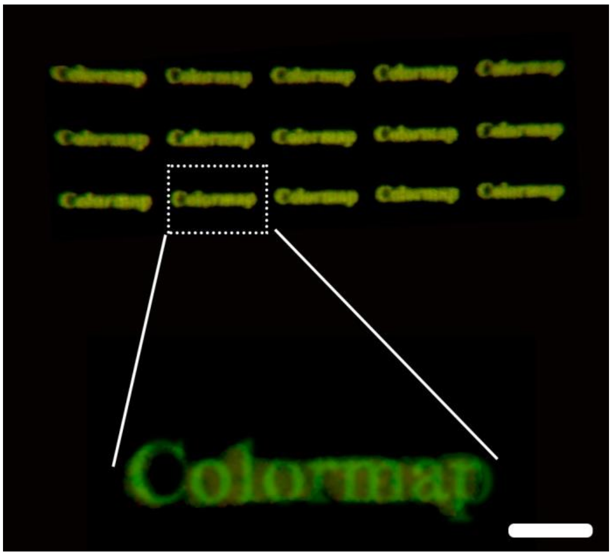

Figure S20. An image of the letters "Colormap" with the point size of 5 printed using g-CDs as ink after spraying with CPPO/H_2O_2 solution. Scale bar = 7 mm. The below letters are the magnified image in the marked area.

Figure S21. The QR code of Zhengzhou University used for printing

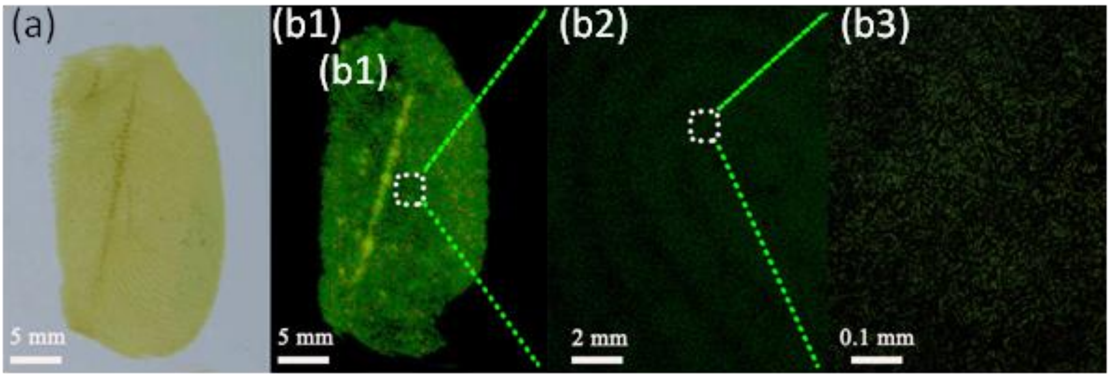

Figure S22. a,b) An image of fingerprint before (a) and after (b1-b3) spraying a mixture solution of CPPO and H_2O_2. (b2) and (b3) are the magnified image in the marked area in (b1) and (b2), respectively.

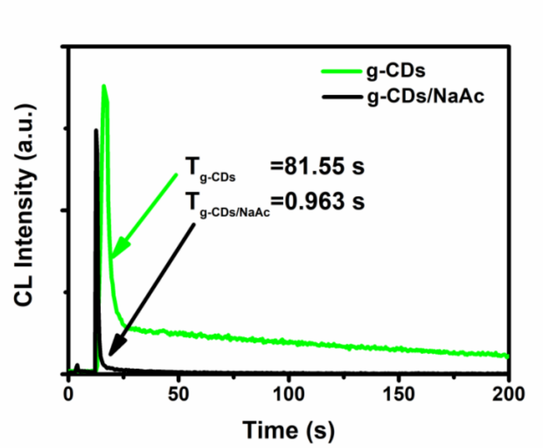

Figure S23. The CL decay of g-CDs and g-CDs/NaAc in the oxalate system on paper. And the lifetime of the g-CDs and g-CDs/NaAc CL system is 81.6 and 0.96 s, respectively**.**

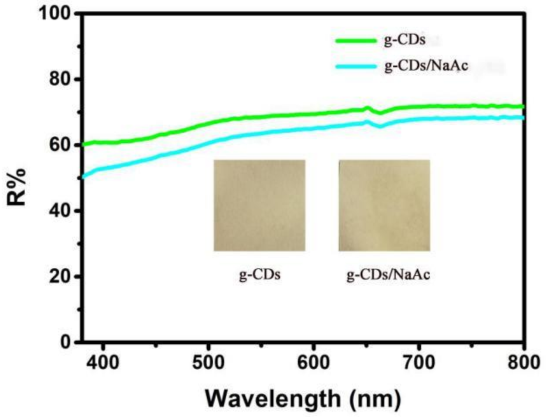

Figure S24. The reflectivity (R%) spectra of g-CDs and g-CDs/Na_2AC on paper. The insets are the typical images of two CDs-based paper under sunlight)

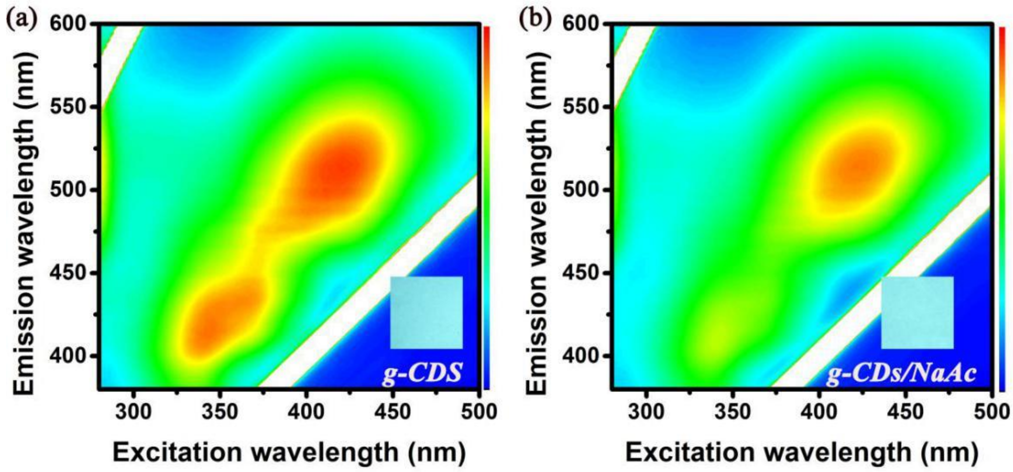

Figure S25. a,b) Excitation-emission matrices of the g-CDs and g-CDs/NaAC on paper. The insets are the fluorescence image of the typical images paper under 365 nm excitation.

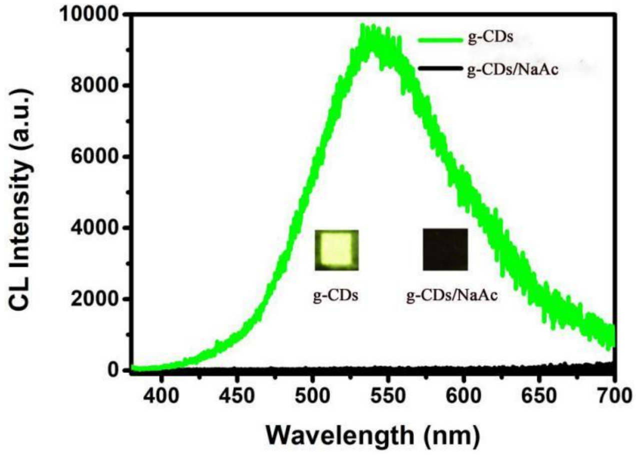

Figure S26. Chemiluminescence spectra of the g-CDs and g-CDs/NaAC on paper after 10 s of spraying the mixture solution of CPPO and H_2O_2 on the paper. The insets are the images of the typical images paper.

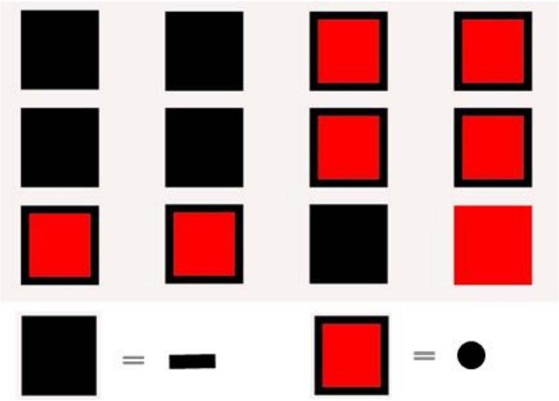

Figure S27. The pattern for information encryption with Morse code.

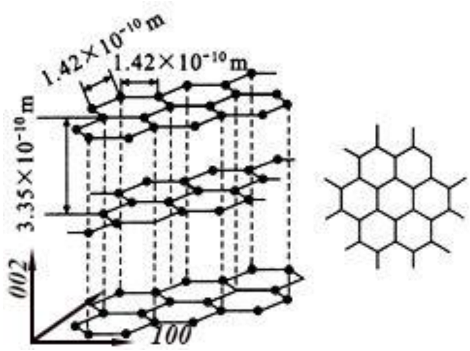

Figure S28. The crystallographic of graphite structure.

Note: As shown in Figure S28, the (100) crystallographic plane is the in-plane of graphitic layers, while (002) is interplanar spacing between two neighboring graphitic layers. In the HRTEM image, it is easy to observe the (100) crystallographic plane due to the stretch of nanosized CDs composed of a few graphitic layers on the copper film. For the powder XRD pattern, the entire information of lattice planes has been included. Due to the stack effect in the aggregated CD powder, the (002) plane may expose and dominate the integral information of the XRD pattern. Actually, similar phenomenon has been observed in other papers (*Adv. Mater.* **2016**, *28*, 3516; *Adv. Mater.* **2018**, *30*, 1704740; *Adv. Opt. Mater.* **2017**, *5*, 1700416; and *Angew. Chem. Inter. Ed.* **2012**, *51*, 12215).

Table S2 The chemiluminescence property of nanomaterial in previous reports

	PL peak [nm]	CL peak [nm]	CL QY [einsteins mol^{-1}]	CL system	Refs
b-CDs	476	468	6.60×10^{-4}	CPPO-CDs-H_2O_2	This work
g-CDs	543	526	2.52×10^{-3}	CPPO-CDs-H_2O_2	This work
r-CDs	634	631	9.32×10^{-3}	CPPO-CDs-H_2O_2	This work
CDs	454 nm	-	-	CDs-$K_2S_2O_8$-TEA	[5]
Gly-CQDs	485 nm	450 nm	-	luminol–KMnO4–CQDs	[6]
CDs	410 nm	425 nm	-	CDs-luminol	[7]
CDs	450 nm	500 nm	-	Ce(IV)-Na2S2O3-CDs	[8]
CDs	450 nm	500 nm	-	CDs/$K_3Fe(CN)_6$	[9]
CTAB@CD	410 nm	530 nm	-	CTAB@CD-Co(II)–H_2O_2–OH^-	[10]
CDs	450 nm	510 nm	-	CDs-$NaNO_2$-H_2O_2	[11]
CDs	525 nm	555 nm	-	CDs-NaOH	[12]
CDs	520nm	440 nm 610 nm	-	CDs-KMnO4	[13]
r-CDs	440 nm	440 nm	-	r-CDs-KMnO4	[13]
CDs	360 nm	490 nm	-	CDs-H_2O_2-HSO_3	[14]
CDs	450 nm	500 nm 650 nm	-	CDs–KMnO4	[15]
CDs	450 nm	500 nm	-	CDs–cerium(IV)	[15]
Au NPs	415 nm	415 nm	(2.8 ±0.3) ×10^{-5}	Au NPs-TCPO-H_2O_2	[16]
Au NPs	415 nm	640 nm	-	Gold colloids-$KMnO_4$-H_2SO_4	[17]
CdTe	550nm -580nm	540 nm -620nm	-	CdTe-Oxidation	[18]

Reference

[1] F. Yuan, Z. Wang, X. Li, Y. Li, Z. Tan, L. Fan, S. Yang, *Adv Mater* **2017**, *29*, 1604436.

[2] X. Zhen, C. Zhang, C. Xie, Q. Miao, K. L. Lim, K. Pu, *ACS Nano* **2016**, 10, 6400.

[3] H. Cui, Z. Zhang, M. Shi, Y. Xu, Y. Wu, *Anal. Chem.* **2005**, 77, 6402.

[4] D. Mao, W. Wu, S. Ji, C. Chen, F. Hu, D. Kong, D. Ding, B. Liu, *Chem* **2017**, 3, 991.

[5] H. Zhang, X. Zhang, and S. Dong. *Anal. Chem.* **2015**, 87, 11167.

[6] Z. Yan, Y. Yu, J. Chen, *Anal. Methods* **2015**, 7, 1133.

[7] Y. Guo, B. Li, *Carbon* **2015**, *82*, 459.

[8] M. Amjadi, J. L. Manzoori, T. Hallaj, M. H. Sorouraddin, *Microchim. Acta* **2014**, 181, 671.

[9] M. Amjadi, J. L. Manzoori, T. Hallaj, M. H. Sorouraddin. S*pectrochim.* A*cta* **2014**, 122, 715.

[10] J. Shi, C. Lu, D. Yan, L. Ma, *Biosens. Bioelectro.* **2013**, *45*, 58.

[11] Z. Lin, W. Xue, H. Chen, J. Lin, *Anal. Chem.* **2011**, *83*, 8245.

[12] L. Zhao, F. Di, D. Wang, L. Guo, Y. Yang, B. Wan, H. Zhang, *Nanoscale* **2013**, *5*, 2655.

[13] P. Teng, J. Xie, Y. Long, X. Huang, R. Zhu, X. Wang, L. Liang, Y. Huang, H. Zheng, *J. Lumin.* **2014**, *146*, 464.

[14] W. Xue, Z. Lin, H. Chen, C. Lu, J. M. Lin. *J. Phys. Chem. C* **2011**, 115, 21707.

[15] Z. Lin, W. Xue, H. Chen, J. Lin, *Chem. Commun.* **2012**, *48*, 1051.

[16] H. Cui, Z. Zhang, M. Shi, Y. Xu, Y. Wu, *Anal. Chem.* **2005**, *77*, 6402.

[17] Z. F. Zhang, H. Cui, M. J. Shi. *Phys. Chem. Chem. Phys.* **2006**, 8, 1017.

[18] Z. Wang, J. Li, B. Liu, J. Hu, X. Yao. *J. Phys. Chem. B* **2005**, 109, 23304.

Supplementary video.

Supplementary Movie 1: This movie depicts the process of b-CD based CL illumination device.

Supplementary Movie 2: This movie depicts the process of g-CD based CL illumination device.

Supplementary Movie 3: This movie depicts the process of r-CD based CL illumination device.
